# A novel cuproptosis-related gene signature for predicting prognosis in cervical cancer

**DOI:** 10.3389/fgene.2022.957744

**Published:** 2022-08-25

**Authors:** Lei Lei, Liao Tan, Long Sui

**Affiliations:** ^1^ Cervical and Vaginal Precancerous Lesion Diagnosis and Treatment, Obstetrics and Gynecology Hospital of Fudan University, Shanghai, China; ^2^ Department of Cardiology, The Third Xiangya Hospital, Central South University, Changsha, China

**Keywords:** cervical cancer, cuproptosis, gene signature, prognosis, tumor immune microenvironment

## Abstract

**Purpose:** Cuproptosis, a form of copper-induced cell death, can be a promising therapeutic target for refractory cancers. Hence, we conducted this research to explore the association between cuproptosis and prognosis in cervical cancer (CC).

**Methods:** For constructing a prognostic signature based on cuproptosis-related genes from TCGA database, the least absolute shrinkage and selection operator Cox regression was utilized. The GSE44001 cohort was utilized for validation.

**Results:** A total of nine cuproptosis-related genes showed distinct expression in CC and normal samples in TCGA-GTEx cohort. Two risk groups were identified based on a seven-gene signature. A significant decrease in overall survival was observed in the high-risk group (*p* < 0.001). The risk score (HR = 2.77, 95% CI = 1.58–4.86) was an autocephalous predictor with a better predictive ability than the clinical stage. Functional analysis indicated that immune activities were suppressed more in the high-risk group than in the low-risk group. A total of 11 candidate compounds targeting the signature were identified.

**Conclusion:** A total of seven cuproptosis-related gene signatures were constructed to predict prognosis and propose a new therapeutic target for patients with CC.

## Introduction

Cervical cancer (CC) is the fourth most common and the fourth leading fatal malignancy in females worldwide, accounting for more than 600,000 newly diagnosed cases and 340,000 death cases around the world in 2020 (Sung et al., 2021). Oncogenic human papillomavirus infection is the major cause of CC (Olusola et al., 2019; Lei et al., 2020). Due to effective human papillomavirus vaccination and high-quality early screening projects, incidence and mortality rates have decreased gradually in recent years (Ronco et al., 2014; Lei et al., 2020). However, because of the insufficient implementation of effective prevention measures, CC remains one of the most common causes of cancer mortality among females in 36 countries, especially developing countries (Sung et al., 2021). Additionally, CC survival has been stagnant since the mid-1970s (Jemal et al., 2017), particularly reflecting the lack of major therapeutic advances for patients with recurrence and metastasis (Siegel et al., 2020). Hence, novel and reliable prognostic biomarker models are crucial to ameliorate the prognosis of CC.

Copper, like other trace metals, is an essential nutrient for cell life. However, it is a double-edged sword whose metabolic disturbances could contribute to critical aspects of cell death and result in some life-threatening diseases, such as Menkes disease and Wilson disease (Bandmann et al., 2015; Czlonkowska et al., 2018). It was reported that patients with Wilson disease had an increased incidence of hepatocellular carcinoma (Kumagi et al., 2004; Thattil and Dufour, 2013), evoking the possibility that aberrant copper accumulation could contribute to tumorigenesis, including CC (Zhang et al., 2018). Although various tumor cells require a higher level of copper (Gupte and Mumper, 2009; Baszuk et al., 2021; Lin and Yang, 2021), a moderately excessive window that focused more on increases of intracellular copper can be applied to selectively kill cancer cells (Ge et al., 2022). Nevertheless, how exactly copper disruption leads to cell death has remained obscure until recently. Tsvetkov et al. (2022) uncovered a previously unidentified mechanism of copper-induced cell death termed as cuproptosis, which is characterized as disturbing specific mitochondrial metabolic enzymes (Tsvetkov et al., 2022), different from traditional apoptosis, necrosis, autophagy, or pyroptosis-mediated cell death (Su et al., 2015). They revealed that overloaded copper could selectively perturb the tricarboxylic acid cycle, leading to lipoylated protein aggregation and subsequent loss of Fe-S cluster protein, resulting in proteotoxic stress and eventually cell death. This finding suggests that cuproptosis could be a promising therapeutic target for refractory cancers (Tsvetkov et al., 2022), whereas the association between cuproptosis and prognosis has not been elucidated in patients with CC.

This research compared the distinct expression level of cuproptosis-related genes between normal cervical and CC samples from The Cancer Genome Atlas (TCGA) and Genotype-Tissue Expression (GTEx) cohorts. Using least absolute shrinkage and selection operator (LASSO) Cox regression, we generated an innovative prognostic signature with a high predictive value for overall survival (OS) in CC patients based on seven identified cuproptosis-related genes. Furthermore, we identified candidate compounds aimed at the cuproptosis-related gene signatures by using the publicly available drug sensitivity database.

## Materials and methods

### Data source

Gene transcriptome and the corresponding clinicopathological and survival data on CC patients were acquired from TCGA database (https://portal.gdc.cancer.gov/repository). Meanwhile, three and ten healthy cervical transcriptome data sets were obtained from the TCGA and GTEx databases (https://xenabrowser.net/datapages/), respectively. All data access and further analysis methods were conducted, following the guidelines of TCGA and GTEx. Before analyses, duplicate samples were deleted from the expression matrix, and ultimately, 306 CC and 13 healthy tissues were retained for further analysis. A total of 13 cuproptosis-related genes (*FDX1*, *LIAS*, *LIPT1*, *GCSH*, *DBT*, *DLST*, *DLD*, *DLAT*, *PDHA1*, *PDHB*, *SLC31A1*, *ATP7A*, and *ATP7B*) were included for subsequent analysis based on the process of cuproptosis described in the previous study (Tsvetkov et al., 2022).

### Bioinformatics analysis

Expression differences of 13 cuproptosis-related genes between tumor and normal tissues were compared by the “limma” package in the R language (version: 4.1.3). mRNA expression levels and their correlation with clinical and pathological characteristics were presented using a heatmap conducted by the “pheatmap” package. The “corrplot,” “igraph,” and “reshape2” packages were used to show the correlation among cuproptosis-related genes. The CC patients were divided into distinct clusters based on consensus expression of cuproptosis-related genes by the “ConsensusClusterPlus” package. For the survival analysis, the “survminer” package was used to delimit the cut-off value. Gene ontology (GO) and Kyoto Encyclopedia of Genes and Genomes (KEGG) annotations were conducted using the “clusterProfiler” package and presented using lollipop plots visualized by the “ggplot2” package.

### Construction of the prognostic signature

To evaluate the prognostic value of cuproptosis-related genes, all cuproptosis-related genes were included in the LASSO Cox regression model (“glmnet” package). We further calculated the risk score for every CC patient. The risk score formula was as follows: Risk Score = ∑Coef_i_ * X_i_ (Coef_i_: the regression coefficient, X_i_: gene expression value). According to the median risk score, the CC patients in TCGA database were clustered into two groups (low-risk and high-risk), and Kaplan–Meier analysis was used to compare OS rates between the two risk groups. Principal component analysis (PCA) based on the selected gene signature was conducted using the “stats” package. The predictive value of the cuproptosis-related gene prognostic signature was evaluated by receiver operating characteristic (ROC) curves of 1-, 3-, and 5-year survival rates and area under the ROC curves (AUCs).

### Gene expression omnibus cohort validation

The mRNA expression matrix and clinical characteristics of the external validation data were acquired from the Gene Expression Omnibus (GEO) database (https://www.ncbi.nlm.nih.gov/geo/, ID: GSE44001). A total of 300 CC patients were enrolled in this cohort. Risk score calculation, PCA, Kaplan–Meier curve analyses, and ROC curves were performed to assess the performance of the cuproptosis-related gene prognostic signature constructed based on the TCGA cohort.

### Potential drugs targeting cuproptosis-related prognostic signature model exploration

To acquire potential drugs based on the cuproptosis-related gene prognostic signature model, the half-maximal inhibitory concentration (IC50) of each drug obtained from the Genomics of Drug Sensitivity in Cancer (GDSC) website was calculated. The “pRRophetic” package was used to predict the IC50 of drugs obtained from the GDSC website during CC patients.

### Validation of the mRNA expression level of cuproptosis-related genes

A total of six frozen cervical squamous cell carcinoma and adjacent normal tissues were collected from the Obstetrics and Gynecology Hospital of Fudan University from 2019 to 2020. Each patient was informed, and the consent form was signed. All of these specimens were pathologically verified. None of the participants received any anti-cancer treatment before the operation. The study was approved by the Ethics Review Board of Obstetrics and Gynecology Hospital of Fudan University (No. 2017-17). Total RNA was isolated from tissues using the TRIzol reagent (Invitrogen, China), according to the manufacturer’s protocol. For cDNA, reverse transcription was performed using the PrimerScript^TM^RT Master Mix (TaKaRa, Japan). Finally, we performed a quantitative reverse transcription-polymerase chain reaction (qRT-PCR) on cDNA using TB Green Premix Ex Taq™ II (TaKaRa, Japan). GAPDH expression functioned as an internal reference. The primers that were used in this research were listed as follows: GAPDH-F: CCA​GGT​GGT​CTC​CTC​TGA, GAPDH-R: GCT​GTA​GCC​AAA​TCG​TTG​T; PDHA1-F: TGG​TAG​CAT​CCC​GTA​ATT​TTG​C, PDHA1-R: ATT​CGG​CGT​ACA​GTC​TGC​ATC; LIPT1-F: TTG​CTA​AAG​AGC​CCT​TAC​CAA​G, LIPT1-R: TCA​TCC​GTT​GGG​TTT​ATT​AGG​TG.

### Statistical analysis

The gene expression levels were compared using a single-factor analysis of variance and paired *t*-test, and categorical variables were compared using the Pearson chi-squared test. The Mann–Whitney U test was applied to compare immune cell infiltration and immune activation between the two risk groups. R software (v4.1.3) was used to perform all statistical analyses.

## Results

### Expression profile, correlation, and interaction of cuproptosis-related genes in cervical cancer

**FIGURE 1:**
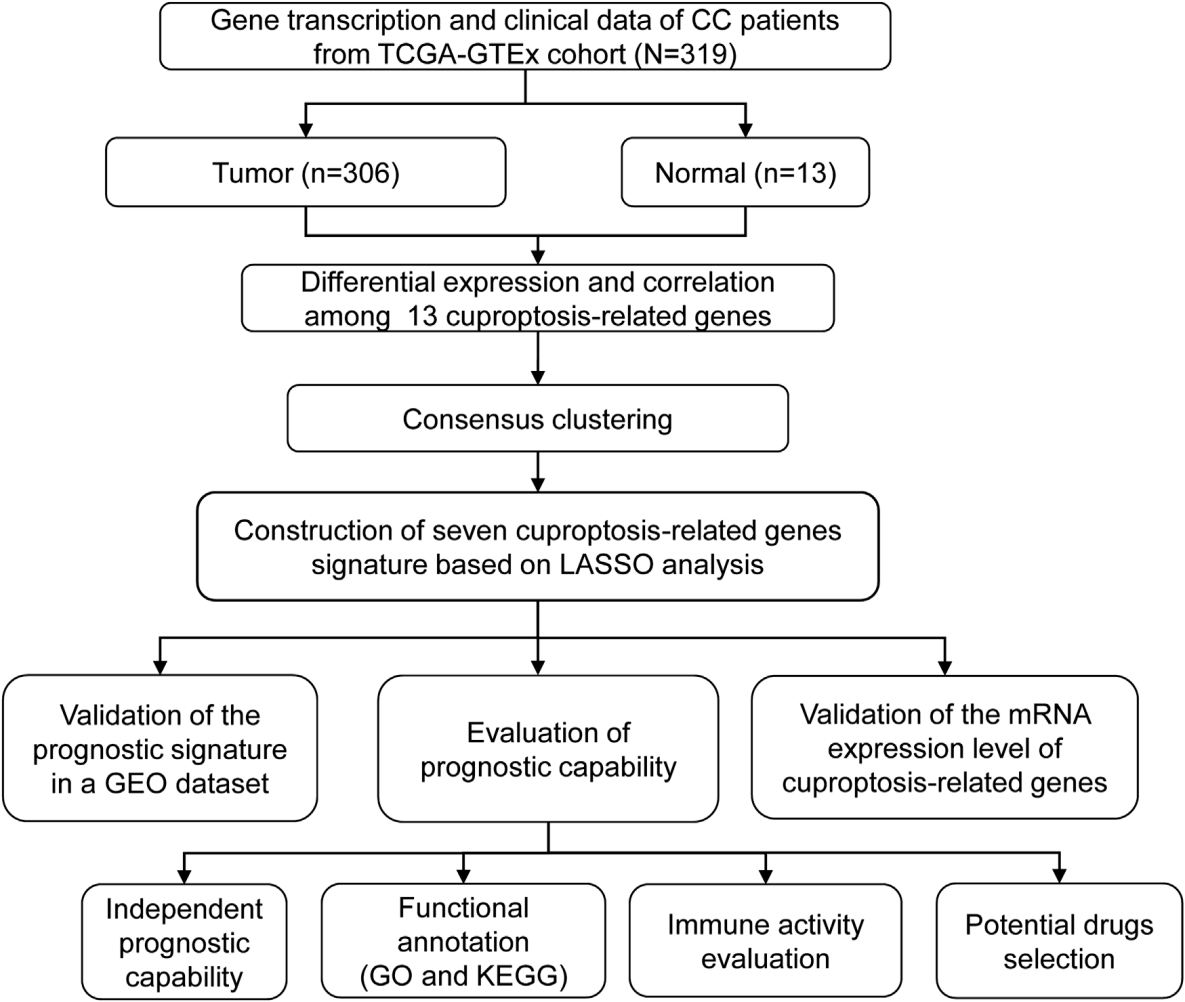
Flow diagram of the research.

**FIGURE 2:**
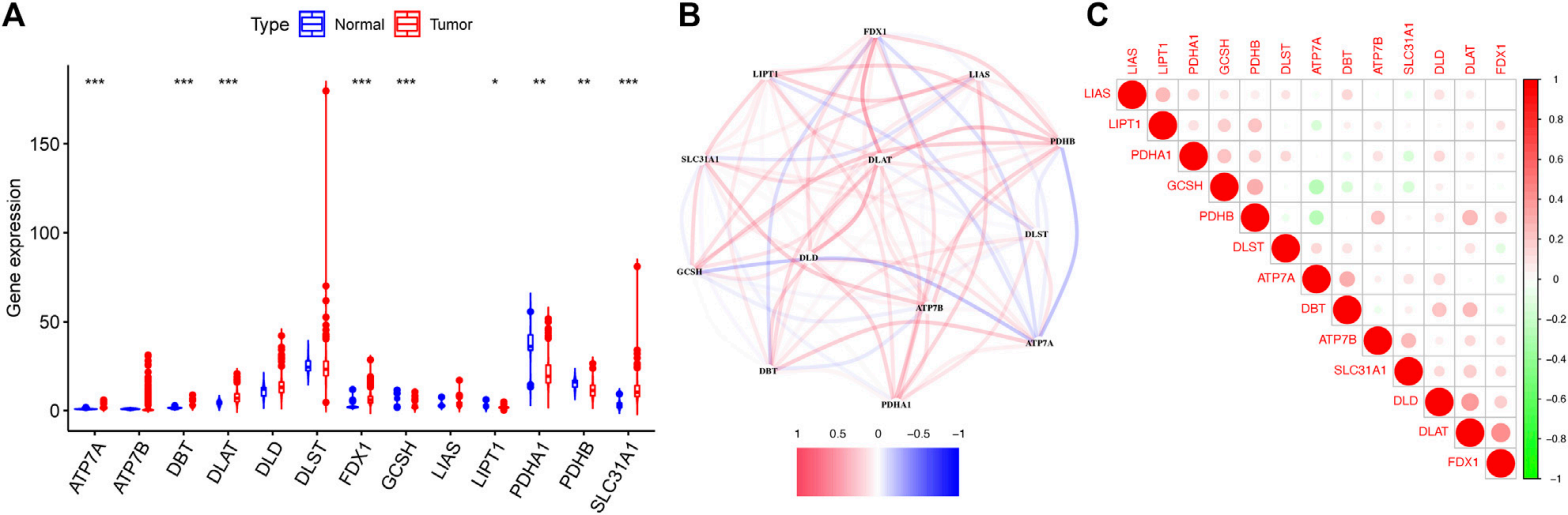
Expression of 13 cuproptosis-related genes in TCGA-GTEx cohort. **(A)** Violin plot presented the distinct expression of cuproptosis-related genes between normal cervical tissues and cervical tumor tissues. **(B)** PPI network showed the interactions among cuproptosis-related genes. **(C)** Correlations among cuproptosis-related genes.

The detailed flow diagram of our research is presented in Figure 1. Transcriptome data from TCGA-GTEx cohort were used to analyze cuproptosis-related gene expression levels. A violin plot illustrated the distinct expression of 13 genes between CC and healthy cervical samples (Figure 2A). A total of five genes (*ATP7A*, *DBT*, *DLAT*, *FDX1*, and *SLC31A1*) showed significantly increased expression levels, while four genes (*GCSH*, *LIPT1*, *PDHA1*, and *PDHB*) showed decreased expression in CC compared with healthy cervical samples (*p* < 0.05).

The protein–protein interactions among 13 cuproptosis-related genes were retrieved *via* the STRING database (Figure 2B), and it revealed that three genes (*DLD*, *DLAT*, and *ATP7B*) were hub genes. We further analyzed the correlations among these genes by Pearson correlation analysis. The results showed that all genes had a weak correlation with each other (Figure 2C).

### Consensus clustering identified two clusters of cervical cancer patients

**FIGURE 3:**
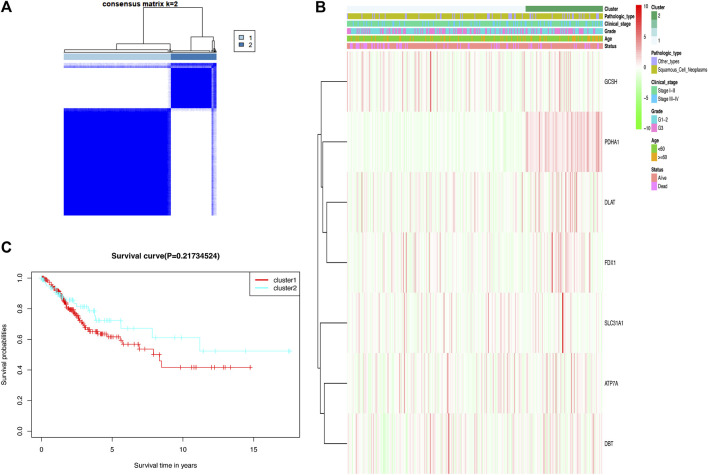
Two clusters based on consensus clustering of cuproptosis-related Genes. **(A)** Consensus clustering matrix for *k* = 2; a total of 306 patients with cervical cancer were grouped into two clusters. **(B)** Clinicopathologic features of the two clusters based on differentially expressed cuproptosis-related genes. **(C)** Kaplan–Meier survival curves for patients in the two clusters.

In the TCGA cohort, 306 CC patients were grouped into several clusters based on the consensus of the mRNA expression for cuproptosis-related differentially expressed genes (DEGs), and *k* = 2 showed the optimal clustering stability from *k* = 2 to 9 based on the expression similarity (Figure 3A). Ultimately, the CC patients were divided into two distinct clusters, namely, cluster 1 and cluster 2. A heat map disclosed that there was no difference in clinicopathological characteristics between the two clusters (Figure 3B). Moreover, there was no significant difference in the OS rate between the two clusters according to Kaplan–Meier curves (Figure 3C).

### Construction of a prognostic signature based on cuproptosis-related genes

**FIGURE 4:**
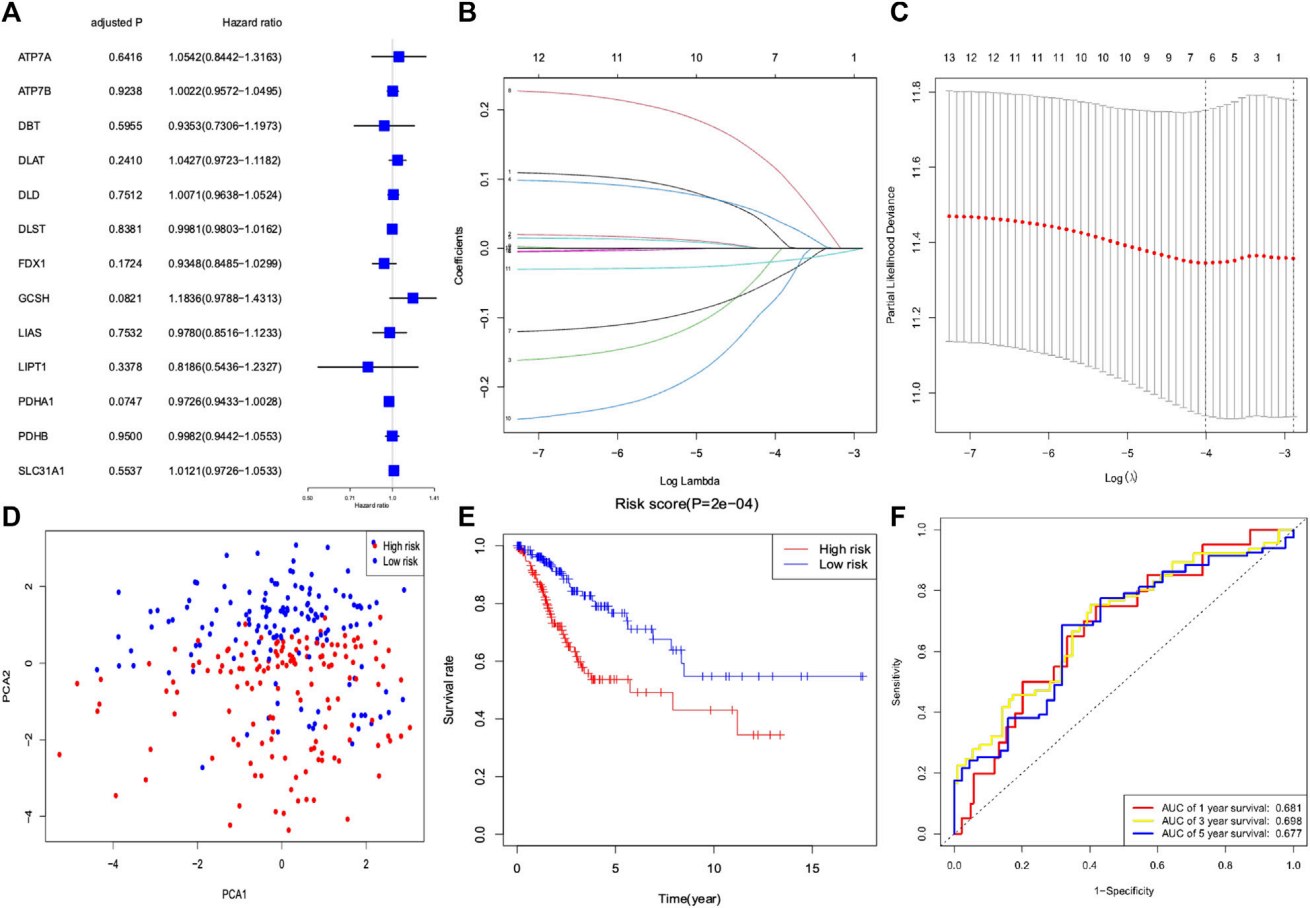
Development of a risk signature for cervical cancer patients collected from TCGA database. **(A)** Univariate Cox regression of cervical cancer for 13 cuproptosis-related genes. **(B)** LASSO regression of 13 cuproptosis-related genes. **(C)** Cross-validation in the LASSO regression for optimizing parameter selection. **(D)** PCA plot for cervical cancer based on the risk score. **(E)** Kaplan–Meier survival curves for overall survival in the two groups. **(F)** AUC values of the ROC curve of the risk score.

**FIGURE 5:**
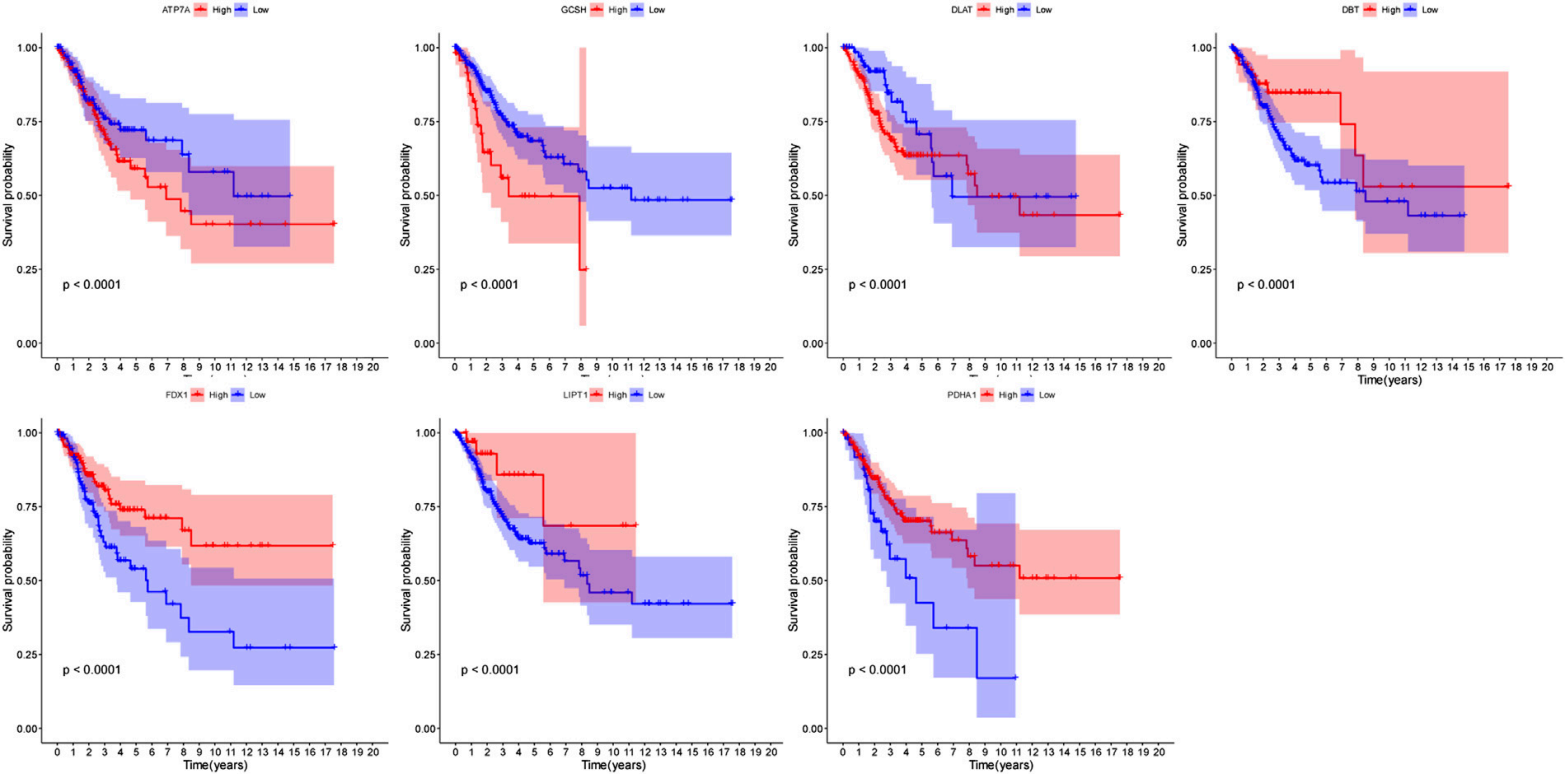
Kaplan–Meier survival analyses for each cuproptosis-related gene of the prognostic signature.

We conducted a univariate Cox regression analysis based on 13 cuproptosis-related genes in CC samples from the TCGA cohort (Figure 4A). The results showed three genes (*GCSH*, *FDX1*, and *PDHA1*), which were related to OS (*p* < 0.2). A LASSO Cox regression algorithm was utilized to construct a risk signature based on the expression of 13 cuproptosis-related genes. A total of seven genes were chosen for the construction of a prognostic signature based on the optimal setting for the tuning parameter *λ* (Figures 4B,C). Kaplan–Meier survival analyses for each cuproptosis-related gene of the prognostic signature are shown in Figure 5. Therefore, the risk score was calculated as follows: Risk Score = 0.1309 * ATP7A- 0.1691 * DBT +0.0999 * DLAT −0.1126 * FDX1 + 0.2140 * GCSH −0.2112 * LIPT1 −0.0293 * PDHA1. According to the median risk score, the CC cohort was then classified into two groups (high-risk and low-risk groups). The two distinct risk groups are shown in the PCA plot (Figure 4D). A significantly poorer OS rate could be observed for the high-risk group than for the low-risk group (Figure 4E). Time-dependent ROC curves demonstrated the prognostic ability of the risk score. The AUC values were 0.681 (1-year), 0.698 (3-year), and 0.677 (5-year) (Figure 4F).

### Validation of the prognostic signature in a gene expression omnibus dataset

**FIGURE 6:**
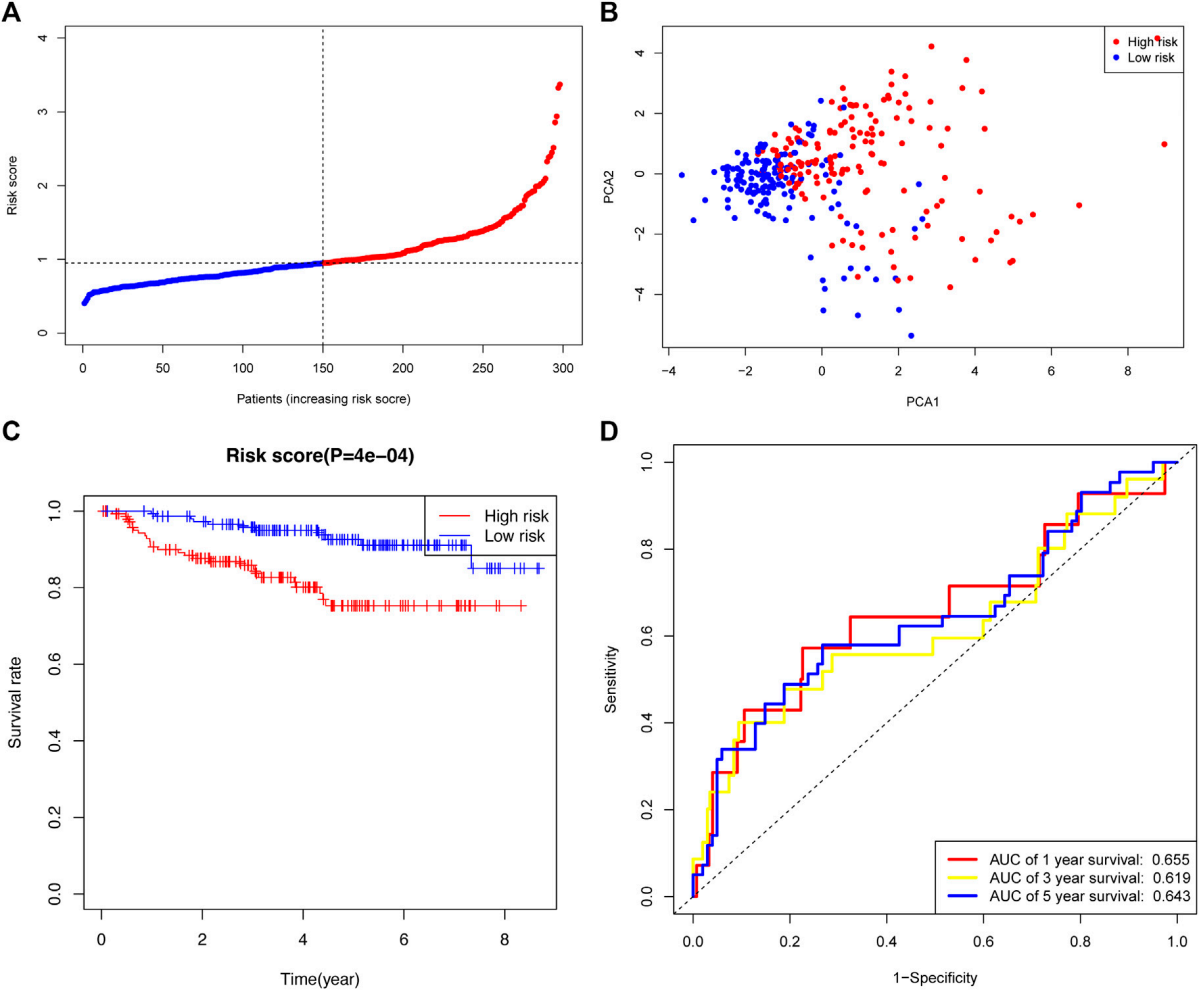
Validation of the risk signature in the GEO cohort. **(A)** Distribution and median risk score in the GEO cohort. **(B)** PCA distribution plot. **(C)** Kaplan–Meier curves of cervical cancer between the two risk groups. **(D)** AUC values of the ROC curve of the risk score in the GEO cohort.

The GSE44001 cohort was used to validate the robustness of the prognostic signature developed by TCGA cohort. The 300 CC patients were also divided into high- or low-risk groups. The distribution of risk scores in the GEO dataset is presented (Figure 6A). The PCA plot showed a differential distribution of the two risk groups (Figure 6B). The high-risk group had a lower OS rate than the low-risk group (Figure 6C). The AUC values of the prognostic signature were 0.655 (1-year), 0.619 (3-year), and 0.643 (5-year) (Figure 6D).

### Independent prognostic value and clinicopathological characteristics of the prognostic signature

**FIGURE 7:**
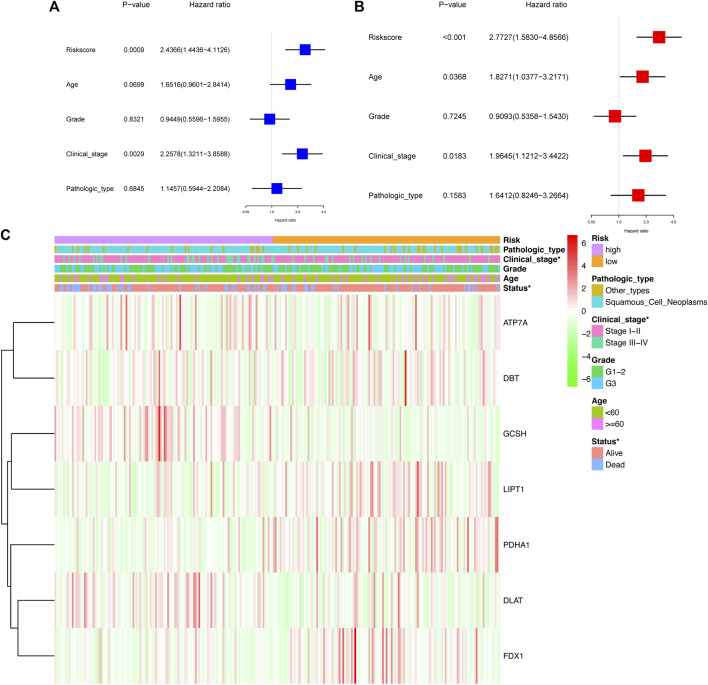
Prognostic value of the risk signature. Univariate Cox analysis **(A)** and multivariate Cox regression **(B)** regarding OS for the risk score in TCGA cohort. **(C)** Heatmap showed the association between clinical and pathological characteristics and in the two groups.

The univariate Cox analysis showed that the risk score and clinical stage had a significant association with OS in CC patients from TCGA cohort (Figure 7A). The multivariate Cox regression model demonstrated that the risk score [Hazard ratio (HR) = 2.77, 95% confidence interval (CI) = 1.58–4.86], age (HR = 1.83, 95% CI = 1.04–3.22), and clinical stage (HR = 1.96, 95% CI = 1.12–3.44) were the autocephalous predictors for CC (Figure 7B). Furthermore, high-risk scores were significantly related to advanced clinical stages and poor survival (*p* < 0.05) (Figure 7C).

### Functional annotation of the prognostic signature

**FIGURE 8:**
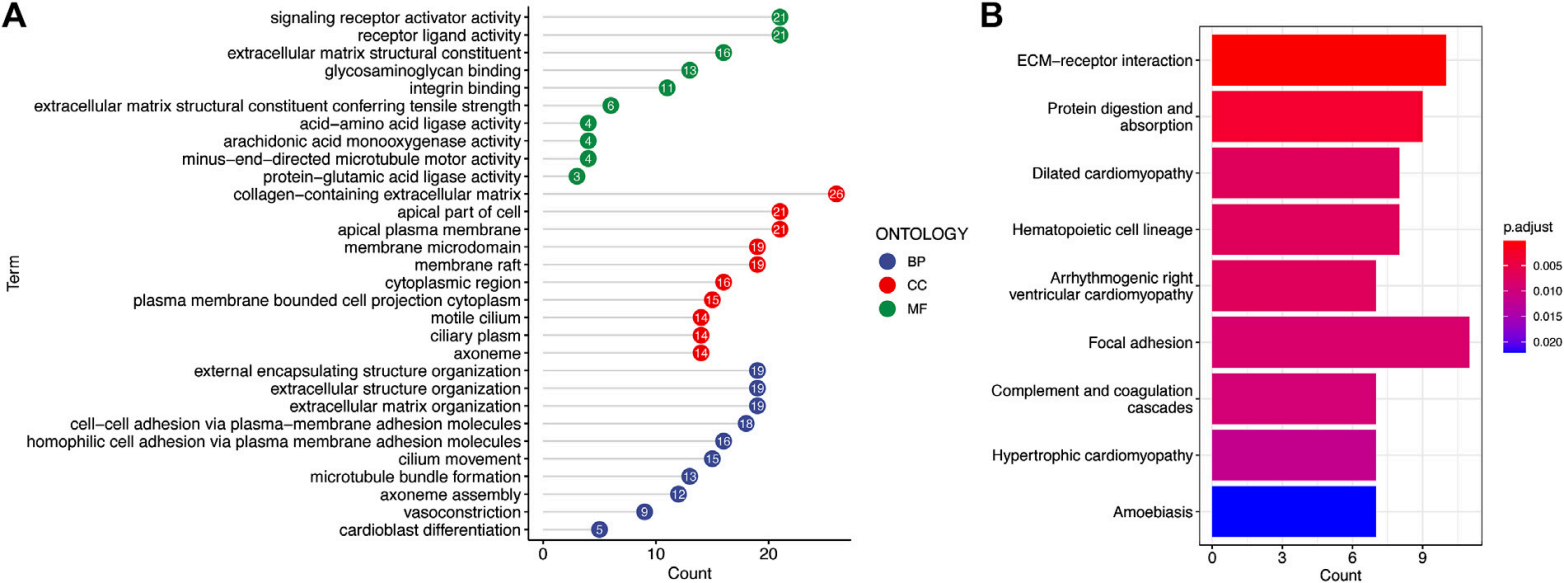
Functional annotation of the low-risk and high-risk groups in TCGA cohort. **(A)** Most significant GO enrichment. **(B)** Result of KEGG pathway enrichment based on the differentially expressed genes between the two risk groups (the longer bars indicate that more genes were enriched, and the deepening red indicates that the differences were more apparent).

To explore potential biological functions and pathways of the signature, GO enrichment and KEGG pathway analyses of DEGs between the two risk groups were conducted. The significant GO enrichment was primarily observed within several biological processes, such as the signaling receptor activity, receptor ligand activity, and extracellular matrix structural constituent (Figure 8A). These DEGs were significantly enriched in focal adhesion and extracellular matrix receptor interaction that are closely correlated with the invasion and metastasis processes of cancer cells based on the KEGG enrichment analysis (Figure 8B).

### Comparison of the immune activity between two risk groups

**FIGURE 9:**
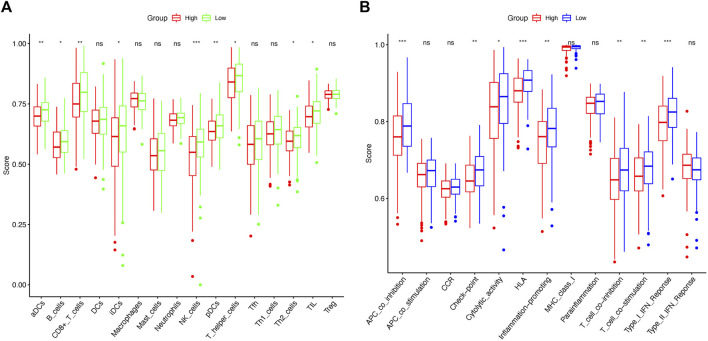
Comparison of ssGSEA scores between the low-risk and high-risk groups in TCGA cohort. Enrichment scores of 16 immune cells **(A)** and 13 immune-related pathways **(B)** between the two risk groups in cervical cancer samples. *p*-values were represented as follows: ns, not significant; *p < 0.05; **p < 0.01; ***p < 0.001.

ssGSEA was used to calculate enrichment scores for 16 types of immune cells and 13 immune-related pathways in order to investigate the immune state between the two risk groups. Remarkably, the results revealed that the proportions for NK cells, aDCs, CD8^+^ T cells, and pDCs differed significantly between the high-risk and low-risk groups (Figure 9A). Furthermore, the high-risk group was associated with decreased APC co-inhibition, HLA, and type I IFN response scores (Figure 9B).

### Identification of potential drugs targeting the prognostic signature

**FIGURE 10:**
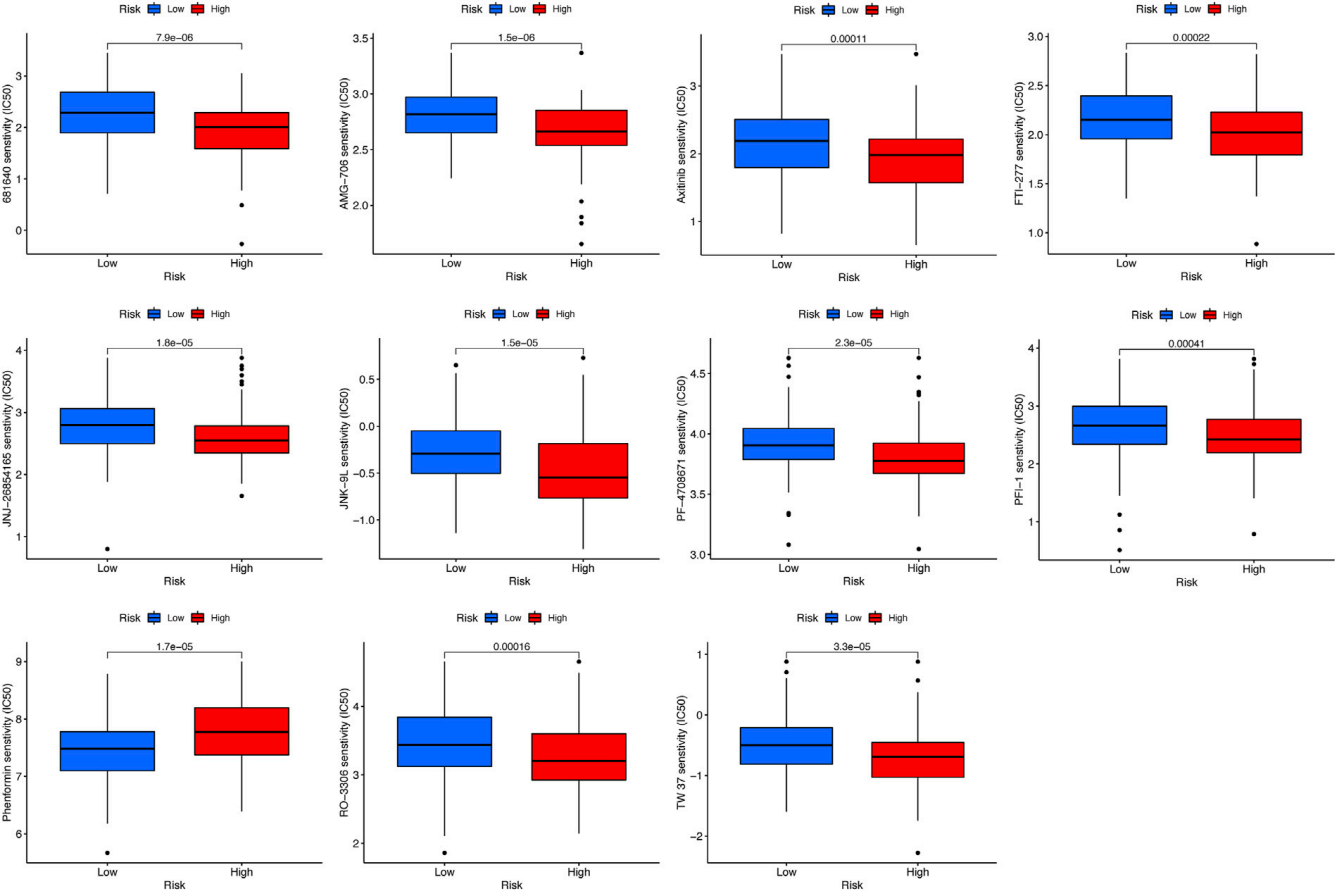
Estimated IC50 levels of 11 compounds between the two risk groups from the GDSC database.

To identify potential drugs targeting the prognostic signature for CC patients, the pRRophetic algorithm was conducted to estimate the therapeutic response based on the IC50 available in the GDSC database. Totally, 11 compounds were screened out for significant differences in the estimated IC50 between the high-risk and low-risk groups, and the high-risk group was more sensitive to all compounds except phenformin (Figure 10).

### Validation of the mRNA expression level of cuproptosis-related genes

**FIGURE 11:**
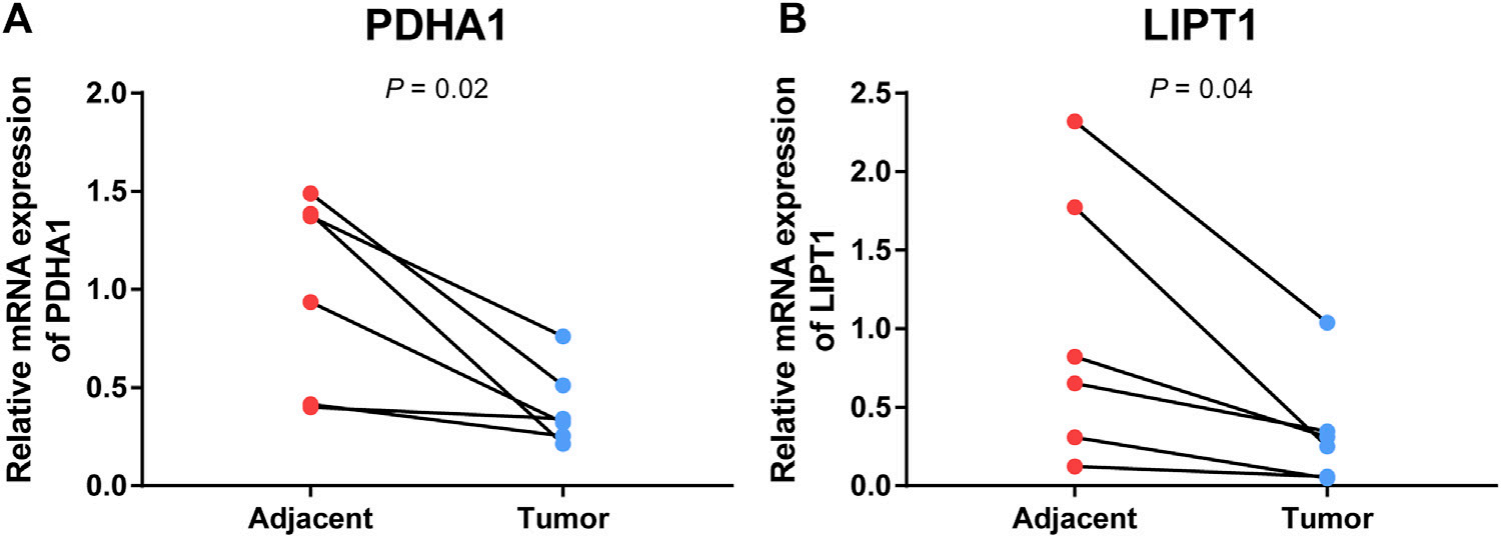
Validation of mRNA expression levels of cuproptosis-related genes in paired tumor tissues. mRNA levels of PDH1 **(A)** and LIPT1 **(B)** in paired tumor tissues were measured by qRT-PCR.

To further validate the prognostic value of cuproptosis-related genes in the signature, qRT-PCR was conducted to compare the gene expression differences between CC and adjacent cervical tissues. The result showed that *PDHA1* and *LIPT1* were significantly downregulated in CC tissues (*p* < 0.05, Figures 11A,B), which was consistent with the result of the previous bioinformatics analysis based on TCGA database.

## Discussion

The prognosis for patients with advanced or metastatic CC remains poor despite recent advances in surgery, chemotherapy, radiotherapy, and immunotherapy (Cohen et al., 2019; Ferrall et al., 2021). Some cancers have been treated with copper ionophores, such as elesclomol. However, these clinical trials have been less successful (Monk et al., 2018; O'Day et al., 2013) because either a sufficient understanding of the drug’s mechanism or a biomarker of the appropriate patient population has remained obscure. Cuproptosis, a recently discovered form of novel cell death, may invigorate studies exploring the use of copper to treat cancer (Kahlson and Dixon, 2022). The new mechanism uncovered by Tsvetkov et al. (2022) suggests that copper ionophore therapy is more effective for tumors that express high amounts of lipoylated tricarboxylic acid enzymes and that undergo mitochondrial respiration. Exploiting copper toxicity, in particular, may provide a promising therapeutic insight for CCs that are resistant to other forms of programmed cell death. However, the association between cuproptosis and prognosis in CC remains unclear.

In our research, we comprehensively analyzed differential expression of 13 currently known cuproptosis-related genes in CC samples and their prognostic values. We constructed a novel cuproptosis-related seven-gene signature by the LASSO Cox regression analysis, which had a significant correlation with the OS in patients with CC. In addition, an external GEO cohort was used for validating the signature. The previous study indicated that *FDX1* could encode a ferredoxin reductase known to reduce Cu^2+^ to more toxic Cu^1+^ and facilitate protein lipoylation (*DBT*, *GCSH*, *DLST*, and specifically *DLAT*). Copper can directly bind and promote the aggregation of lipoylated protein. On the other hand, *FDX1* causes the loss of iron-sulfur cluster proteins. Lipoylated protein aggregation and loss of Fe-S cluster proteins are the fundamental mechanisms of cuproptosis. Additionally, cuproptosis is positively regulated by *LIPT1* and *PDHA1*. *ATP7A* is considered to inhibit cuproptosis by decreasing intracellular Cu levels (Tsvetkov et al., 2019; Tsvetkov et al., 2022). In this prognostic signature, *DBT*, *FDX1*, *LIPT1*, and *PDHA1* acted as positive predictors, while *ATP7A*, *DLAT*, and *GCSH* acted as negative predictors for survival in CC patients. Kaplan–Meier survival curves indicated that the higher risk scores were related to worse prognosis among CC patients, so we could reasonably infer those patients in the high-risk group who were less susceptible to cuproptosis.

Although the role of cuproptosis in tumorigenesis and tumor development has remained unclear, recent studies have highlighted the fact that most of the seven cuproptosis-related genes in this signature play a vital role in the development of cancer. Consistent with our study, the decreased expression of *FDX1* was associated with a poor survival outcome in lung adenocarcinoma. Mechanistically, knockdown of *FDX1* mainly affected the energy metabolism, whose reprogramming is a characteristic of carcinoma (Hanahan and Weinberg, 2011), but showed no apparent effect on cell proliferation and apoptosis in lung adenocarcinoma cells (Zhang et al., 2021). Tsvetkov also found that *FDX1* was a direct target of elesclomol, which promotes copper-dependent cell death in cancer cells (Wang et al., 2002). DLAT, encoding a subunit of the mitochondrial pyruvate dehydrogenase complex (Wang et al., 2002), was increased in acute myeloid leukemia compared with normal peripheral blood cells (Shan et al., 2014). Goh et al. (2015)also discovered that *DLAT* was upregulated in gastric cancer cells and played a vital role in proliferation and carbohydrate metabolism in gastric cancer cells. Interestingly, *PDHA1*, another subunit of pyruvate dehydrogenase complex, seemed to play a double-edged sword role in human tumors. Low expression of PDHA1 is associated with poor OS in ovarian cancer and gastric cancer (Li et al., 2016; Song et al., 2019), whereas PDHA1 plays the oncogenic role in prostate tumor and cholangiocarcinoma by regulating lipid biosynthesis and the Warburg effect, thus modulating cancer energy metabolism reprogramming (Chen et al., 2018; Dan et al., 2018). In breast cancer tissue and cells, GCSH protein was overexpressed, and GCSH could strengthen the viability of cancer cells and thus result in tumorigenesis, which was congruent with our result (Adamus et al., 2018). ATP7A, a copper efflux transporter, has also been reported to promote tumorigenesis and metastasis, be associated with poor survival, and enhance resistance to platinum drugs in various cancer types, including CC (Samimi et al., 2003; Shanbhag et al., 2019; Arnesano and Natile, 2021; Yu et al., 2021). Hence, *ATP7A* is a potential therapeutic target to selectively enhance the effectiveness of platinum-based chemotherapy (Ishida et al., 2010; Gao et al., 2021).

Multivariate Cox analysis further demonstrated the risk score was an autocephalous prognostic factor that had a better predictive ability than the clinical stage. Additionally, the risk score was significantly associated with the clinical stage in CC. These results indicated that the inactivated process of cuproptosis was associated with the advanced clinical stage and unfavorable survival in CC patients.

Afterward, using the GO and KEGG analyses, we revealed the DEGs between the two risk groups were related to invasion and metastatic processes of cancer cells, so it is reasonable to speculate that cuproptosis is related to invasion and metastasis in CC patients. In addition, the high-risk group had universally lower immune cell counts and activities than the low-risk group, such as NK cells, CD8+ T cells, and type I IFN response, which could suppress tumor proliferation and metastasis (Henning et al., 2018; Hodgins et al., 2019; Liang et al., 2021). Hence, we can reasonably assume that cuproptosis is significantly associated with anti-tumor immunity in CC, and improved anti-tumor immunity activities combined with the utilization of copper toxicity in CC patients at high risk has the potential to improve the prognosis. Eventually, our study first identified 11 potential anticancer drugs targeting the prognostic signature using the GDSC database.

Our results brought new opinions to the evaluation and therapy of CC. First, it is time to construct a prognostic model on the basis of cuproptosis-related genes. Cuproptosis, differing from any known mechanisms of cell death, is defined as a novel type of cell death reliant on mitochondrial respiration. Such an unusual mechanism may bring up new solutions for the treatment of cancer. The significant performance of our prognostic model suggested that cuproptosis could play a critical role in regulating the progress of CC. Moreover, the difference of the immune status between the high-risk and low-risk groups indicated that cuproptosis-related genes could impact the immune response during CC. Finally, potential anticancer drugs targeting the prognostic signature predicted in our study provided a new orientation for anti-cancer drug discovery.

Unavoidably, there are some limitations to our research. First, we retrieved data retrospectively from TCGA and GEO databases. The cuproptosis-related gene prognostic signature needs to be further verified with prospective, multicenter, and real-world data. Second, the underlying mechanisms of cuproptosis and associated genes in CC need further investigation. Third, the function of potential drugs targeting the prognostic signature needs further in-depth studies.

Overall, our research constructs a signature based on seven cuproptosis-related genes for predicting the prognosis of CC patients, and the signature may facilitate novel research directions in investigating the mechanisms of cuproptosis and personalized prognostic predictions and serve as a new therapeutic target for CC patients.

## Data Availability

The datasets presented in this study can be found in online repositories. The names of the repository/repositories and accession number(s) can be found in the article/Supplementary Material.
